# Cytokine and metabolic regulation of adipose tissue Tregs

**DOI:** 10.1097/IN9.0000000000000013

**Published:** 2022-11-01

**Authors:** Cody Elkins, Chaoran Li

**Affiliations:** 1 Department of Microbiology and Immunology, Emory University School of Medicine, Atlanta, GA, USA; 2 Immunology and Molecular Pathogenesis Graduate Program, Graduate Division of Biological and Biomedical Sciences, Emory University Laney Graduate School, Atlanta, GA, USA

**Keywords:** VAT Treg, obesity, metabolism, inflammation, cytokine

## Abstract

Since their discovery over a decade ago, much has been learned regarding the importance and function of visceral adipose tissue (VAT)-resident regulatory T cells (Tregs). VAT Tregs play a critical role in controlling VAT inflammation and alleviating metabolic disease. However, this population is disrupted in obesity which exacerbates VAT inflammation and metabolic abnormalities. Therefore, understanding the factors governing the accumulation and maintenance of VAT Tregs, both at steady state and under disease conditions, is crucial for identifying the mechanisms underlying obesity-associated metabolic disease and developing novel therapies. Expansion and maintenance of the VAT Treg compartment is strongly influenced by factors in the local tissue microenvironment, including cytokines, T-cell receptor ligands, hormones, and various metabolites. This mini-review will primarily focus on recent advances in our understandings regarding the regulation of mouse epididymal VAT (eVAT) Tregs, which are the most thoroughly characterized VAT Treg population, by tissue microenvironmental factors and cellular metabolic processes. We will also briefly discuss the limited knowledge available regarding the regulation of mouse ovarian VAT (oVAT) Tregs and human omental VAT Tregs, highlight some lingering questions, and provide a prospective view on where the field is heading.

## 1. Introduction

Regulatory T cells (Tregs) play a critical role in controlling both sterile inflammation and inflammation in response to antigen challenge ^[1]^. However, Tregs are far from a homogeneous population of cells. Tregs exist in a variety of tissues including the adipose tissue, the central nervous system, lung, liver, skin, muscle, and intestines. In general, compared to their lymphoid organ counterparts, these “tissue-Tregs” exhibit a more activated and effector-like phenotype, upregulating various activation markers (eg, CD44, CD69) and suppressive molecules (eg, CTLA-4, IL-10). However, Tregs from each tissue also acquire a unique set of gene signatures that facilitate their maintenance and promote their specific functions in different tissue microenvironments. These aspects of tissue Tregs have been summarized in a few recent reviews ^[2–4]^.

One particular tissue-Treg population of interest is that resident in the visceral adipose tissue (VAT) ^[5]^. Identified by Feuerer et al over a decade ago, VAT Tregs, particularly those in the epididymal VAT (eVAT) of male mice, have been found to be critical for modulating the eVAT inflammatory milieu and maintaining systemic metabolic homeostasis. However, this population is significantly reduced during obesity ^[6–10]^. Multiple studies show that induced expansion of eVAT Tregs can alleviate obesity-induced inflammation and promote insulin sensitivity in mice fed a long-term high fat diet (HFD), indicating that disruption of eVAT Tregs is indeed a major driver for obesity-induced metabolic abnormalities ^[7,10–12]^. Of note, one study also suggests that the accumulation of eVAT Tregs in old mice (>1 year old) promotes aging-associated insulin resistance, indicating that eVAT Tregs may have different functions in different contexts ^[13]^.

VAT Tregs are unique from their lymphoid tissue counterparts in several respects (Table 1). First, eVAT Tregs compose a large fraction of the CD4^+^ T-cell compartment. Compared to lymphoid tissue Tregs, which compose ~10% to 20% of CD4^+^ T cells, eVAT Tregs start to accumulate at 12 to 16 weeks of age and can make up to 80% of the CD4^+^ T cell population in the eVAT by 20 to 30 weeks of age ^[7]^. Second, T-cell receptor (TCR) sequencing of eVAT Tregs shows a unique and clonally expanded TCR repertoire that has minimal overlap with lymphoid-tissue Tregs, indicating that eVAT Tregs recognize and expand in response to specific local antigen(s) ^[7,14,16,18]^. Third, eVAT Tregs exhibit a unique transcriptional program, including upregulation of certain surface markers such as killer cell lectin-like receptor G1 (KLRG1) and suppression of tumorigenicity 2 (ST2; the receptor for the cytokine IL-33), several chemokine receptors such as CCR2, and most interestingly, a set of enzymes involved in lipid metabolism. The distinct transcriptome is mainly driven by the transcription factor peroxisome proliferator-activated receptor gamma (PPARγ), the master regulator of lipid storage and adipocyte differentiation ^[11]^. In addition to this major PPARγ^+^ population, several additional Treg sub-populations were recently found to also present in eVAT (see sections below). Last, VAT Tregs show strong sex differences driven by sex hormones. In contrast to those in the epididymal VAT depot (eVAT) from male mice, Tregs do not accumulate in female fat depots such as ovarian VAT (oVAT), and oVAT Tregs are phenotypically more similar to lymphoid-tissue Tregs ^[19]^. While VAT Tregs are important for controlling the local tissue inflammatory environment, this modulation is not a one-way street. A variety of factors existing in the local tissue environment, most notably cytokines, antigens, hormones, and various metabolites, can directly and/or indirectly control VAT Treg accumulation and phenotypic acquisition. Furthermore, cellular metabolic pathways may also control VAT Treg expansion and effector function. Here, we discuss our current understanding on these topics and discuss important questions that remain to be addressed which are summarized in Table 2.

**Table 1:** Comparison of mouse lymphoid and eVAT Tregs.

Characteristic	Lymphoid Tregs	eVAT Tregs
Frequency of CD4^+^ T cells	10%–20%	60%–80% (20–30 weeks old)
TCR repertoire	Diverse TCR repertoire	Clonally Restricted with enrichment of particular TCRs (eg vTreg53) ^[14,15]^
Transcriptional program	Foxp3-driven transcriptional program	• PPARγ and Foxp3-driven transcriptional program ^[11]^ • Also depend on Blimp1, BATF, and IRF4 ^[12]^
Markers	• Mostly Foxp3^+^ PPARγ^−^ • Small fraction of Foxp3^+^ PPARγ^lo^ (VAT Treg precursors) ^[15,16]^	3 main sub-populations: ^•^ Foxp3^+^, PPARγ^+^, ST2^hi^, CD73^lo^, KLRG1^+^^[5,7,17]^ • Foxp3^+^, PPARγ^−^, ST2^lo^, CD73^hi^ ^[17]^ • Foxp3^+^, PPARγ^−^, CD73^lo^, ST2^lo^, CXCR3^+^^[15]^

PPARγ: peroxisome proliferator-activated receptor gamma, TCR: T-cell receptor, Treg: regulatory T cell, eVAT: epididymal visceral adipose tissue.

**Table 2:** Summary of key findings and remaining questions.

Regulatory factors	Key findings and remaining questions
IL-33/ST2 signaling	*Key findings:* • IL-33 can expand VAT Tregs directly ^[12,14,20]^ or indirectly ^[21]^ • Direct IL-33 signaling is largely dispensable for accumulation of VAT Tregs at steady state ^[22]^ *Questions remaining:* Under what contexts is Treg-intrinsic IL-33 signaling important for VAT Tregs?
Inflammatory cytokines (TNFα, IFNγ, IFNα)	*Key findings:* • In mice, TNFα and IFNγ suppress VAT-Treg accumulation indirectly ^[21,23]^, while IFNα derived from pDCs directly reduces survival and proliferation of VAT Tregs through IFNAR1 signaling ^[9]^ • In humans, IFNγ reduces VAT-Treg suppressive function by promoting expression of inhibitory molecule PD-1 ^[24]^ *Questions remaining:* • What is the molecular mechanism by which IFNα disrupts VAT Tregs? • Do other obesity-associated inflammatory molecules such as IL-1β and IL-6 disrupt VAT Treg homeostasis and function?
TCR-antigen recognition	*Key findings:* • VAT Tregs exhibit a clonal expanded TCR repertoire ^[7,14]^ • TCR specificity is required, but not sufficient, for VAT Treg accumulation ^[15]^ • VAT Tregs depend on MHCII-restricted ligands ^[14]^ • Several surrogate peptides have been identified as ligands of a VAT Treg clone, vTreg53 ^[25]^ *Questions remaining:* What are the endogenous antigens recognized by VAT Treg TCRs?
Sex hormones	*Key findings:* • VAT Tregs from gonadal fat depot of male and female mice differ in terms of frequency, number, and phenotype ^[15,19]^ ^•^ Sex dimorphism of VAT Tregs was mostly driven by sex hormones via indirect mechanisms ^[19]^ *Questions remaining:* Are sex difference seen in mouse VAT Tregs translatable to human omental fat Tregs?
Metabolism and other factors	*Key findings:* ^•^ VAT Tregs show increased expression of genes involved in lipid metabolism relative to their lymphoid counterparts ^[11]^ ^•^ Insulin promotes differentiation of PPARγ^+^ CD73^lo^ ST2^hi^ VAT Tregs ^[17]^ *Questions remaining:* What cellular metabolic pathways do VAT Tregs depend on?

PPARγ: peroxisome proliferator-activated receptor gamma, TCR: T-cell receptor, Treg: regulatory T cell, VAT: visceral adipose tissue.

## 2. Cytokines

### 2. 1. IL-33/ST2

Compared to their lymphoid-tissue counterparts, eVAT Tregs highly express ST2 (encoded by *Il1rl1*), the receptor for IL-1 family cytokine IL-33. In light of this, the importance of the IL-33/ST2 axis is one of the most thoroughly characterized to date with respect to eVAT Tregs. IL-33 is an alarmin typically associated with a type 2 immune response ^[26]^. In eVAT, the expression of IL-33 is largely restricted to particular sub-populations of PDGFRα^+^Sca-1^+^ mesenchymal stromal cells (mSCs), with a small contribution from a population of mesothelial cells ^[27,28]^. IL-33 is retained in the nucleus of these cells until it is released upon some trigger of cellular stress or cell death. Following release, IL-33 induces expansion and promotes effector function of ST2-expressing cells such as type 2 innate lymphoid cells (ILC2s), T helper 2 (T_H_2) cells, and Tregs.

IL-33 has a major impact on eVAT Treg accumulation. Both in vitro and in vivo treatments with recombinant IL-33 (rIL-33) were shown to selectively expand ST2^+^ eVAT Tregs and, in the latter case, also alleviate HFD-induced inflammation and metabolic abnormalities ^[12,14,20]^. However, the relative importance of direct vs. indirect effects of IL-33 on eVAT Treg accumulation and function was still a matter of debate ^[29]^. Initial studies found that ST2 global knockout mice had significantly reduced eVAT Tregs at steady state ^[12,14]^. Additionally, mixed bone marrow (BM) chimera models using donor BM deficient for ST2 or its signaling adaptor, MyD88, showed a significant reduction in eVAT Tregs among cells derived from mutant BM compared to those derived from wild type (WT) BM ^[12]^. Furthermore, Treg-specific ablation of ST2 in vTreg53 TCR (an expanded eVAT Treg clone) transgenic mice led to a significant reduction of clonotype^+^ KLRG1^+^ eVAT Tregs ^[15]^. These results, along with the ability for rIL-33 to induce expansion of purified eVAT Tregs in vitro, suggested that IL-33 may directly promote accumulation of eVAT Tregs.

However, these experiments did not preclude the possibility that other non-Treg cells could contribute to IL-33-induced expansion of eVAT Tregs. One such mediator is ILC2s. This was initially shown by Molofsky et al where they deleted IL-5 expressing cells (presumably ILC2s) using an IL-5cre-driven model and observed significant impairment of eVAT Treg accumulation at steady state and following rIL-33 treatment ^[21]^. However, there is a possibility that low levels of IL-5 expression in eVAT Tregs could impose confounding, direct effects on eVAT Tregs in this model ^[4]^. More recently, Hemmers et al specifically deleted ST2 and MyD88 in Tregs using a *Foxp3*^*YFPcre*^ model and found no defect in total eVAT Treg accumulation at steady state, although there is a reduction in KLRG1^+^ subsets in *Il1rl1*^*f/f*^*Foxp3-Cre* mice compared to *Il1rl1*^*f/+*^*Foxp3-Cre* mice following rIL-33 treatment. Instead, cell intrinsic ST2 signaling seemed to be especially important for type 2 cytokine production from eVAT Tregs and their ability to limit accumulation of local γδT cells ^[22]^.

Overall, these data support the idea that while IL-33 can directly expand eVAT Tregs, it is largely dispensable for the accumulation of total eVAT Tregs at steady state. However, certain sub-populations of eVAT Tregs (Table 1) could still depend on cell intrinsic ST2 signaling, and a defect in Treg-intrinsic IL-33 signaling may therefore impact the composition of the eVAT Treg compartment rather than overall eVAT Treg abundance. A comprehensive single-cell transcriptomic analysis of eVAT Tregs from *Il1rl1*^*f/f*^*Foxp3-Cre* and *Il1rl1*^*+/+*^*Foxp3-Cre* mice should be able to address this issue.

### 2. 2. Inflammatory cytokines (TNFα, IFNγ, and IFNα)

HFD-induced obesity leads to elevated levels of many inflammatory cytokines in eVAT, notably TNFα, IFNγ, and IFNα. Generally speaking, these cytokines impede eVAT Treg expansion. However, the mechanisms by which this occurs varies.

TNFα and IFNγ appear to inhibit eVAT Treg homeostasis mostly through indirect mechanisms. Co-administration of TNFα or IFNγ with IL-33 suppresses IL-33-induced eVAT Treg expansion in vivo ^[9]^. Conversely, antibody-mediated blockade of TNFα in obese mice enhances eVAT Treg expansion in vivo in response to immunization with eVAT Treg TCR-specific surrogate peptide FAT1562 ^[25]^. Furthermore, YETI mice that have a systemic increase in IFNγ level show a significant reduction in eVAT Tregs ^[21]^, while adipocyte specific ablation of MHCII leads to a reduction in IFNγ levels and an increase in eVAT Tregs ^[30]^. However, treatment of purified eVAT Tregs with TNFα or IFNγ did not impact their proliferation or survival in vitro ^[9]^. These data suggest that TNFα and IFNγ most likely impair eVAT Treg accumulation indirectly. Indeed, in a recent study, Lin and colleagues show that TNFα stimulates the expression and secretion of soluble ST2 (sST2) from adipocytes, which acts as a decoy receptor that inhibits IL-33 signaling and therefore disrupts eVAT Treg homeostasis ^[23]^. Similarly, Locksley and colleagues show that IFNγ inhibits ILC2 activation, which is required for eVAT Treg accumulation via ICOSL-ICOS interactions ^[21]^. Of note, although TNFα does not directly affect proliferation and/or survival of eVAT Tregs, it does lead to phosphorylation of serine 273 of PPARγ and induces a gene signature that resembles what is seen in eVAT Tregs from obese mice ^[8]^.

On the other hand, IFNα plays a more direct role in promoting eVAT Treg loss in obesity ^[9]^. RNA-Seq analysis shows that levels of interferon stimulated genes (ISGs) are significantly increased in eVAT Tregs following long-term HFD feeding, corresponding to their loss in vivo. In addition, antibody-mediated blockade of IFNAR1 or Treg-specific deletion of *Ifnar1* attenuates the loss of eVAT Tregs and improves insulin sensitivity in obese mice. Moreover, administration of IFNα inhibits IL-33-induced eVAT Treg expansion in vivo, and treatment of purified eVAT Tregs with IFNα in vitro also significantly reduced their proliferation and survival. These results suggest that obesity-induced type I IFN directly impairs eVAT Treg expansion. Finally, loss of eVAT Tregs in obesity is preceded by the expansion of IFNα-expressing plasmacytoid dendritic cells (pDCs) in the eVAT, and depletion of pDCs also improves eVAT Treg homeostasis and metabolic indices in obese mice. The molecular mechanism by which IFNα disrupts eVAT Tregs and whether IFNβ has a similar impact on eVAT Tregs in obesity remain to be determined.

## 3. TCR-antigen recognition

The accumulation and phenotype of eVAT Tregs also critically depend on TCR signaling and specificity. TCR sequencing analysis reveals that eVAT Tregs exhibit a less diverse TCR repertoire compared to their lymphoid tissue counterparts and that there is selective expansion of particular Treg clones in eVAT ^[7,14]^, suggesting that antigen recognition plays a key role in promoting eVAT Treg accumulation. In support of this notion, transgenic mice made to express a eVAT Treg associated TCR (vTreg53) exhibit elevated levels of eVAT Tregs, reduced eVAT inflammation, and improved insulin sensitivity at steady state ^[15]^. Moreover, vTreg53 clonotype positive (vTreg53^+^) Tregs from lymphoid tissues preferentially accumulate in eVAT following adoptive transfer into WT recipient mice while clonotype negative (vTreg53^−^) Tregs do not. However, antigen-specificity alone is not sufficient for eVAT Treg accumulation, as vTreg53^+^ CD4^+^ Foxp3^−^ T conventional cells (Tconvs) fail to accumulate in the eVAT following adoptive transfer. Indeed, Foxp3 is shown to be critical for eVAT Treg accumulation ^[15]^.

Despite a clear importance of TCR-specificity in eVAT Treg accumulation, the antigens that eVAT Treg TCRs recognize remain unknown. While successful screenings of Treg-specific peptide antigens have been performed, these screenings were typically on a select group if antigens such as tumor-associated neo-antigens, thymic MHCII presented peptides, or AIRE-dependent protein antigens, and narrowing the number of possible candidate peptides that activate eVAT Treg TCRs remains a challenge ^[25,31–33]^. It was initially speculated that lipid-based antigens were likely candidates. However, global deletion of lipid antigen-presenting MHC-like molecule CD1d had little impact on eVAT Treg accumulation in vivo ^[14]^. On the contrary, eVAT Tregs were found to most closely associate with MHCII-expressing CD11b^−^ CD11c^+^ antigen-presenting cells and global deletion or antibody blockade of MHCII significantly reduced eVAT Tregs ^[14]^. This suggests that eVAT Treg TCRs recognize MHCII presented peptide ligands. Still, the sheer quantity of host peptide candidates presentable to eVAT Tregs, even within the eVAT MHCII peptidome, provides a challenge for efficient screening of eVAT Treg TCR ligands. Using a peptide-MHC yeast library screening system, Fernandes et al identified a number of synthetic MHCII-restricted surrogate peptides (SPs) that bind to and stimulate the vTreg53 transgenic TCR with varying degrees of potency ^[25]^. Determining the core peptide sequence required for activating the vTreg53 TCR should facilitate the identification of the host antigens that eVAT Tregs recognize.

In addition to promoting eVAT Treg accumulation, TCR signaling also plays an important role in controlling the phenotype of these cells. TCR stimulation induces upregulation of *PPARγ*, a key driver for the eVAT Treg transcriptional program. Mechanistically, TCR signaling activates the transcription factors BATF and IRF4, which bind to the *PPARγ* and *Il1rl1* loci and promote their transcription. As a result, *Batf*^*−/−*^ and *Irf4*^*−/−*^ splenic Tregs fail to upregulate these genes in vitro following TCR activation and *Batf*^*−/−*^ and *Irf4*^*−/−*^ mice show a near complete lack of eVAT Tregs while the frequency of splenic Tregs is only mildly affected ^[12]^.

In summary, these data point to an important role for TCR-antigen recognition in supporting eVAT Treg accumulation and acquisition of their distinct phenotype. However, whether TCR is still required after establishment of the eVAT Treg compartment remains unclear. Another area worth exploring is the impact of obesity on TCR signaling in eVAT Tregs. For example, obesity may alter the expression, processing, or presentations of local antigens or modulate the sensitivity of eVAT Treg TCRs. Therefore, understanding whether and how eVAT Treg TCR signaling is impacted in obese conditions is also an important topic of exploration.

## 4. Hormones and sex differences in the VAT Treg compartment

The majority of previously stated findings pertain to male epididymal VAT (eVAT) Tregs. The corresponding gonadal fat depot in females, the ovarian VAT (oVAT) shows reduced appearance of Tregs compared to eVAT at steady state ^[15]^. Moreover, RNA-seq reveals that oVAT Tregs do not show upregulation of typical eVAT-Treg markers such as *PPARγ* and *Il1rl1* (ST2) but are rather more like their splenic counterparts ^[19]^.

The difference in the VAT Treg compartment between sexes can be explained, at least in part, by differences in sex hormones ^[19]^. Androgen receptor deficient (*Ar*^*−/−*^) male mice show reduced ST2^+^ VAT Tregs, while o-estrogen receptor α deficient (*Era*^*−/−*^) female mice show increased VAT Tregs compared to wildtype sex-matched controls. Conversely, in vivo testosterone treatment expands VAT Tregs in female mice and o-estrogen treatment reduces VAT Tregs in male mice. However, the impact of sex hormone signaling on VAT Treg accumulation is mostly indirect, as Treg-specific deletion of androgen receptor has minimal impact on VAT Treg levels, and female WT chimeric mice receiving both WT and *Era*^*−/−*^ BM show no difference in accumulation between WT and *Era*^*−/−*^ VAT Tregs.

The indirect effects of sex hormones on VAT Tregs may occur through several mechanisms. First, hormones may alter VAT Treg accumulation by impacting IL-33-producing VAT MSCs and IL-33 availability ^[19,27]^. Indeed, eVAT shows higher frequencies of IL-33- producing MSCs compared to oVAT. Moreover, *Ar*^*−/−*^ male and *Era*^*−/−*^ female mice show altered VAT MSC cellular composition compared to wildtype controls and rIL-33 injection can expand ST2-expressing oVAT Tregs in vivo, suggesting that IL-33 may be a limiting factor for oVAT Treg accumulation. Second, hormones may affect chemokine-mediated Treg recruitment to the VAT ^[19]^. Compared to oVAT, eVAT expresses significantly higher levels of CCL2, a ligand of CCR2, which is important for recruitment of Tregs into VAT. This effect is mostly attributed to different levels of estrogens, rather than androgens between male and female mice.

Compared to male mice, female mice are less prone to HFD-induced weight gain, VAT inflammation, and metabolic syndrome ^[34]^. Tregs are generally attracted to inflamed sites to suppress inflammation. Therefore, the enrichment of VAT Tregs in males is thought to be a feedback response to elevated basal levels of inflammation in eVAT. Indeed, following long-term HFD feeding, Tregs also increase in the more inflamed oVAT in female mice ^[35]^. The precise impact of estrogen signaling on VAT Treg phenotype and function in obese conditions remains to be determined.

## 5. PPARγ-dependency and VAT Treg metabolism

A defining feature of eVAT Tregs is expression of the transcription factor peroxisome proliferator-activated receptor gamma (PPARγ), which is expressed in over 80% of eVAT Tregs in lean mice over 20 weeks old ^[15]^. PPARγ is essential for eVAT Treg accumulation, and it also drives expression of a significant portion of the eVAT Treg gene signature genes, such as KLRG1, CCR2, and GATA3 ^[11]^. Treg-specific ablation of PPARγ dramatically reduces eVAT Treg levels, and those remaining are more transcriptionally similar to lymphoid-tissue Tregs. Conversely, retroviral-mediated overexpression of Foxp3 and PPARγ in naive, activated CD4^+^ T cells can largely mimic the eVAT Treg gene signature. Co-immunoprecipitation assays also show that Foxp3 and PPARγ interact following co-transduction into HEK293T cells, suggesting these two transcription factors may cooperate to drive the eVAT Treg phenotype. Finally, anti-diabetic drug Pioglitazone, a known PPARγ ligand, significantly expands eVAT Tregs and improves insulin sensitivity in obese mice. However, these effects were attenuated in mice lacking PPARγ specifically in Tregs. These data point to an important role for PPARγ in promoting eVAT Treg expansion and in curtailing metabolic disease.

PPARγ is also known as the “master regulator” of adipocyte differentiation, and eVAT Tregs show increased expression of genes involved in the uptake, synthesis, and storage of lipids (eg, CD36, *Hmgcr*, *Dgat1*, *Pcyt1a*) compared to lymphoid-tissue Tregs. However, whether and how eVAT Tregs rely on these specific lipid metabolic pathways and their dependency on other key metabolic pathways such as glycolysis, fatty acid oxidation (FAO), and oxidative phosphorylation (OXPHOS) are unknown. In vitro-generated Tregs (iTregs) and peripherally derived Tregs (pTregs) have been shown to depend on FAO and OXPHOS, while activated thymus-derived Tregs (tTregs) seem to rely more on the glycolytic-lipogenic pathway ^[36]^. Low numbers of VAT Tregs prevent the use of conventional means for assessing cellular metabolism such as mass spectrometry and seahorse assays. Recent developments in single-cell-based metabolic approaches may facilitate the understanding of metabolic dependencies of eVAT Tregs ^[37]^. For example, flow cytometry has now been widely used to quantify uptake of fluorescent-labeled metabolites such as glucose and various lipids, and to assess mitochondria mass and membrane potential. Mass cytometry (CyTOF) and single-cell RNA-Seq have been used to predict cell metabolic state based on expression of key metabolic regulatory enzymes and transporters ^[38–40]^. Last, Argüello et al has recently developed a flow cytometry-based assay named Single Cell ENergetIc metabolism by profiIng Translation inHibition (SCENITH), which uses puromycin incorporation to assess translational activity and ATP production of individual cells ^[41]^. These single-cell-based approaches will be extremely useful for determining the metabolic and energetic dependency of a rare population like VAT Tregs.

## 6. Other factors

Several additional factors have recently been found to regulate eVAT Treg accumulation and phenotype. Notably, through single-cell ATAC sequencing and paired single-cell RNA and TCR sequencing analysis, Li et al identified two distinct subsets of Tregs from pooled visceral and subcutaneous adipose tissues: CD73^hi^ST2^lo^ Tregs, which are more transcriptionally similar to lymphoid-tissue Tregs, and CD73^lo^ST2^hi^ Tregs, which highly express PPARγ. Interestingly, they reported that insulin signaling plays a key role for the transition from CD73^hi^ST2^lo^ Tregs into CD73^lo^ST2^hi^ Tregs through an HIF-1α-Med23-PPARγ axis ^[17]^. A third population of ST2^−^CD73^−^CXCR3^+^ eVAT Tregs have also been identified through additional single cell analyses, and these cells have been reported to differentiate in response to Th1-associated IFNγ production ^[15,42]^ Additionally, Mittelsteadt et al found that deletion of ICOS significantly increased Tregs in the VAT, but not in subcutaneous adipose tissue, skin, lung, or spleen, suggesting a unique role for ICOS signaling in suppressing VAT Treg expansion ^[43]^. Mechanistically, they propose that loss of ICOS promotes accumulation of VAT Tregs through promoting CCR3 expression.

## 7. Tregs in human omental fat

Human omental fat (the major fat depot in humans) (omVAT) also harbors a distinct population of Tregs. However, there is limited knowledge regarding the regulation of Treg homeostasis in omVAT. Initial studies observed a negative correlation between Foxp3 expression in omVAT and body mass index (BMI) in obese individuals, suggesting omVAT Tregs are lost during obesity ^[7]^. Wu et al subsequently confirmed these findings by observing lower omVAT Tregs with increasing BMI by flow cytometry. Interestingly, they also observed omVAT Tregs highly expressed *PPARG* (PPARγ), consistent with findings in mice, but observed no detectable expression of ST2^[44]^. However, the lack of available adipose tissue samples from lean control patients precluded a proper comparison between healthy and obese omVAT Tregs. Recently, Bradley et al compared VAT Tregs from lean and obese volunteers and indeed found a significant reduction in the levels of VAT Tregs in obese patients, and this reduction corresponded to increased insulin resistance. Moreover, they found increased expression of interferon gamma receptor 1 (*IFNGR1*) in obese VAT Tregs compared to lean controls, and showed that IFNγ reduced Treg suppressive function in vitro by promoting expression of inhibitory molecule PD-1. This suggests that IFNγ may suppress VAT Treg function in obesity by promoting PD-1 expression and an exhausted phenotype ^[24]^.

## 8. Perspective

**Figure 1.**
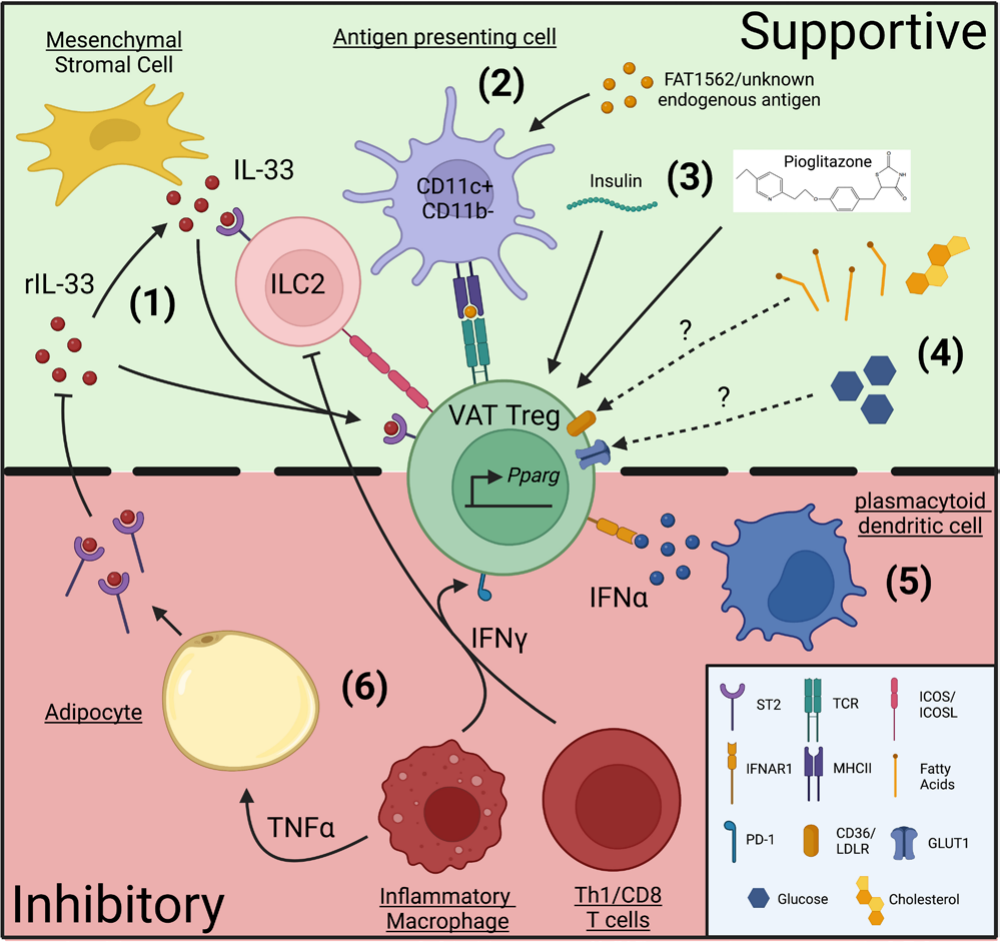
Signals that control VAT-Treg accumulation and phenotype. There are various factors in the adipose tissue microenvironment that regulate VAT-Treg accumulation and phenotype. (1) IL-33 derived from adipose MSCs can bind ST2 on ILC2s which interact with VAT Tregs via ICOS/ICOSL to support VAT-Treg expansion. In certain contexts, VAT Tregs may also respond to IL-33 directly. (2) CD11c^+^ CD11b^−^ dendritic cells present currently unknown endogenous antigens to VAT Tregs to promote their activation and expansion. Synthetic peptide agonist (FAT1562) can also selectively stimulate and expand a VAT Treg TCR clone, vTreg53. (3) Insulin signaling promotes maturation of PPARγ^+^ VAT Tregs, which can be activated by pioglitazone. (4) VAT Tregs exhibit increased expression of lipid transporters (eg, *Cd36*, *Ldlr*) compared to lymphoid-tissue Tregs, but whether VAT Tregs critically rely on lipids or other metabolites such as glucose for proper accumulation and function remains to be addressed. (5) Type I interferon producing pDCs accumulate in VAT during obesity and directly inhibit VAT Tregs survival and proliferation. (6) Other inflammatory cytokines such as TNFα and IFNγ mostly inhibit VAT Tregs indirectly. TNFα induces expression and release of soluble ST2 (sST2) from adipocytes, which can sequester IL-33 to impair ILC2 and VAT-Treg accumulation. IFNγ limits IL-33-induced ILC2 activation, which hinders ILC2-mediated expansion of VAT Tregs, and also impairs human VAT Treg suppression by promoting PD-1 expression. PPARγ: peroxisome proliferator-activated receptor gamma, TCR: T-cell receptor, Treg: regulatory T cell, VAT: visceral adipose tissue.

It is important to keep in mind that the various signals mentioned in this review do not act independently from one another, and that VAT Tregs experience a combination of microenvironmental cues in vivo which together regulate VAT Treg homeostasis (Figure 1). Therefore, understanding how these signals are integrated to support the accumulation and phenotype of VAT Tregs will be crucial for developing combination-based therapies in contexts where these signals are imbalanced (ie, obesity). For example, a combination of TCR activation, IL-33 stimulation, and blockade of pro-inflammatory cytokines might be necessary to efficiently restore/reinvigorate VAT Tregs and improve metabolic indices in obese individuals. As more is learned about the metabolic dependency of VAT Tregs, it will also be worth exploring how these environmental signals influence VAT-Treg metabolism and how cellular metabolism can, in turn, control the response of VAT Tregs to different environmental factors.

Finally, the majority of work to date regarding the modulation of VAT Treg homeostasis has focused on those located in the gonadal fat depot (eVAT for male and oVAT for female) as they are the most enriched and best characterized in mice. However, other adipose depots (perirenal, mesenteric, and subcutaneous) also contain Tregs, which may have a different phenotype and function. Given that PPARγ^+^ Tregs are also present in human omVAT, it will be important to explore whether the many findings regarding the modulation of gonadal fat Treg homeostasis in mice are translatable to Tregs in other mouse-adipose-tissue compartments or to Tregs in human omental fat.

## Conflicts of interest

The authors declare they have no conflicts of interest.

## Funding

This work was supported by the National Institute of Diabetes and Digestive and Kidney Diseases of the National Institutes of Health under Award Number R01DK128061.
